# Programmed death ligand 1 expression in early stage, resectable non-small cell lung cancer

**DOI:** 10.18632/oncotarget.26529

**Published:** 2019-01-15

**Authors:** Manolo D’Arcangelo, Armida D’Incecco, Claudia Ligorio, Stefania Damiani, Maurizio Puccetti, Sara Bravaccini, Luigi Terracciano, Chiara Bennati, Gabriele Minuti, Silvia Vecchiarelli, Lorenza Landi, Marina Milesi, Alberto Meroni, Sara Ravaioli, Maria Maddalena Tumedei, Matteo Incarbone, Federico Cappuzzo

**Affiliations:** ^1^ AUSL della Romagna, Department of Oncology-Hematology, Ravenna, Italy; ^2^ University Hospital of Siena, Medical Oncology and Immunotherapy, Center for Immuno-Oncology, Siena, Italy; ^3^ University of Bologna, DIMES, Pathology, Bologna, Italy; ^4^ AUSL della Romagna, Department of Pathology, Ravenna, Italy; ^5^ Istituto Scientifico Romagnolo per lo Studio e la Cura dei Tumori IRCSS, Bioscences Laboratory, Meldola, Italy; ^6^ University Hospital Basel, Institute of Pathology, Basel, Switzerland; ^7^ Clinica San Carlo, Service of Pathology, Paderno Dugnano, Italy; ^8^ RCCS MultiMedica, Thoracic Surgery, Sesto S.G., Italy

**Keywords:** PD-L1, NSCLC, prognosis, tumor grading

## Abstract

**Introduction:**

For several years non-small cell lung cancer (NSCLC) has been considered non-immunogenic. Recent advances in antitumor immunity brought to the discovery of checkpoints that modulate immune response against cancer. One of them is programmed death receptor 1 (PD-1) and its ligand (PD-L1). Although PD-L1 expression seems predictive of response to anti-PD-1/PD-L1 agents, its prognostic value is unclear. In this study we investigated the prognostic value of PD-L1 expression and its correlation with clinical-pathological characteristics in a cohort of surgically resected NSCLC.

**Material and methods:**

PD-L1 expression was evaluated in 289 surgically resected NSCLC samples by immunohistochemistry. Our cohort included patients not exposed to adjuvant chemotherapy. PD-L1 status was defined as: 1) PD-L1 high (tumor proportion score, TPS≥50%), PD-L1 low (TPS 1-49%), PD-L1 negative (TPS<1%); 2) PD-L1 positive (TPS≥50%) and negative (TPS<50%); 3) as a continuous variable.

**Results:**

Patients were mostly males (79%), former or current smokers (81%), with a median age of 67 years, non-squamous histology (67.5%) and high-grade tumors (55%). PD-L1 tumors were 18.7%. There was no significant association with sex, age, smoking status and histology. A strong correlation between high PD-L1 expression and tumor grade was detected. The difference in median OS in the different groups of patients was not statistically significant.

**Conclusion:**

PD-L1 is not prognostic in surgically resected NSCLC. The association with tumor differentiation suggests that grading could represent an easy-to-assess tool for selecting subjects potentially sensitive to immunotherapy warranting further investigations.

## INTRODUCTION

Lung cancer is the most common cause of cancer-related deaths worldwide. It accounts for 14% of new diagnoses and for 1 in 4 cancer deaths. It is usually diagnosed in advanced stage and survival of metastatic patients remains disappointing [1]. Lung cancer is a heterogeneous disease, including two major histologic categories: Small-Cell Lung Cancer (15-20% of cases) and Non-Small-Cell Lung Cancer (NSCLC, 80-85% of cases). The latter includes adenocarcinoma (60%), squamous cell carcinoma (25%), large cell carcinoma (10%) and other less common histologic subtypes [2]. Deeper understanding of NSCLC biology has recently brought to notable improvement in its treatment thanks to the introduction of target agents for tumors with specific oncogene aberrations. Despite these advances, prognosis of patients with lung cancer remains poor especially for those who do not harbor a driver mutation.

For a long time lung cancer has been considered as non-immunogenic for the lack of activity of therapeutic approaches with cytokines, although the wide T-cell infiltration observed in pathological samples [3, 4]. The discovery of immune checkpoints as regulators of immune response has granted new important insights for the understanding of antitumor immunity. Under normal conditions immune checkpoints are responsible for the balancing of pro- and anti-immune signals within immune cells, allowing the physiological mechanism of self-tolerance [5]. Tumor cells are able to hijack immune-modulatory mechanisms thus ensuring evasion of the immune system surveillance and tumor growth and survival [6]. Some of these mechanisms include reduced expression of the major histocompatibility complex molecules, loss of tumor antigens, production of immunosuppressive mediators and expression of inhibitory checkpoint ligands [7]. Several checkpoints have been identified so far and among them one of the most important is the programmed death receptor 1(PD-1)/programmed death ligand 1 (PD-L1) axis.

PD-L1 (CD247 or B7-homolg 1, B7-H1) is a type I transmembrane glycoprotein and the principal ligand of PD-1. It is widely expressed on dendritic cells, lymphocytes, macrophages, mast cells, endothelial and epithelial cells [8]. Furthermore, expression of PD-L1 occurs in several tumors, including lung cancer [9-40]. Two anti-PD-1 agents, nivolumab and pembrolizumab, and one anti-PD-L1 agent, atezolizumab, have been approved over the last three years for the treatment of metastatic NSCLC and other agents are under investigation. In the Keynote 024 study, pembrolizumab effectively prolonged survival of NSCLC patients expressing PD-L1 in at least 50% of tumor cells compared to platinum-based chemotherapy in first-line setting [41-42]. Conversely, in the CheckMate 026 study nivolumab failed to produce a survival benefit compared to chemotherapy in first-line setting even in the subgroup of individuals with high levels of PD-L1 expression [43]. However, nivolumab significantly prolonged survival compared to docetaxel in second-line setting irrespective of PD-L1 levels [44-45]. A plethora of clinical trials are evaluating the combination of immunotherapy with chemotherapy, target therapies or other immune-directed agents; other trials are exploring immunotherapy in the neoadjuvant and adjuvant settings [46-48]. Although PD-L1 expression seems predictive for sensitivity to anti-PD-1/PD-L1 strategies, its prognostic impact remains unclear in early as well as in advanced disease [9, 10, 12, 13-15, 17, 19, 21-23, 25-28, 35-40].

In this study we aimed to investigate the prognostic value of high levels of PD-L1 expression and its association with clinical and pathological features in a cohort of surgically resected NSCLC patients not exposed to adjuvant chemotherapy.

## RESULTS

### Patient characteristics

The characteristics of patients are shown in Table 1. The majority of patients were male (79%) and the median age was 67 years (range: 23-89). About one third of tumors were classified as having squamous differentiation (31.1%) based on the immunohistochemistry (IHC) positive staining for p40 and about half of the tumors were poorly differentiated (55%). Although data on smoking exposure were available for a small percentage of cases, the majority of patients with a complete smoking history were smokers at the time of lung cancer surgery (81.6%). Our cohort included patients treated with radical surgery before chemotherapy became a standard treatment and before the approval of any target therapy. For such reason no information on epidermal growth factor receptor (*EGFR*), anaplastic lymphoma kinase (*ALK*) or ROS protoncogene 1 (*ROS-1*) status was available.

**Table 1:** Patients characteristics

	Total	%
**Total no. of patients**	289	100
Median age, years (range)	67 (23-87)	
**Gender**		
Male	229	79
Female	60	21
**Histology**		
Adenocarcinoma	187	64.7
Squamous	90	31.1
Other	12	4.2
**Grading**		
1	14	4.8
2	107	37
3	148	51.2
Not defined	20	7
**Disease stage (TNM 7**^th^**ed)**		
I	80	27.7
II	70	24.2
IIIA	76	26.3
IIIB	5	1.7
Not defined	58	20.1
**Smoking status**		
Available/not available	49/240	17/83
Smoker/Non-smoker	40/9	81.6/18.4

### PD-L1 expression and correlation to clinical-pathological characteristics

Among the 289 tumors analyzed for this study, 176 (60.9%) were negative for PD-L1 expression on tumor cells (tumor proportion score, TPS <1%), 59 (20.4%) showed only weak staining (TPS 1-49%) and 54 (18.7%) were strongly positive (TPS ≥ 50%). The PD-L1 score obtained from the two cores of the same tumor was concordant in most cases (97.9%). In case of discordance (2.1%), the final score was obtained calculating the average value of the two scores.

**Figure 1:**
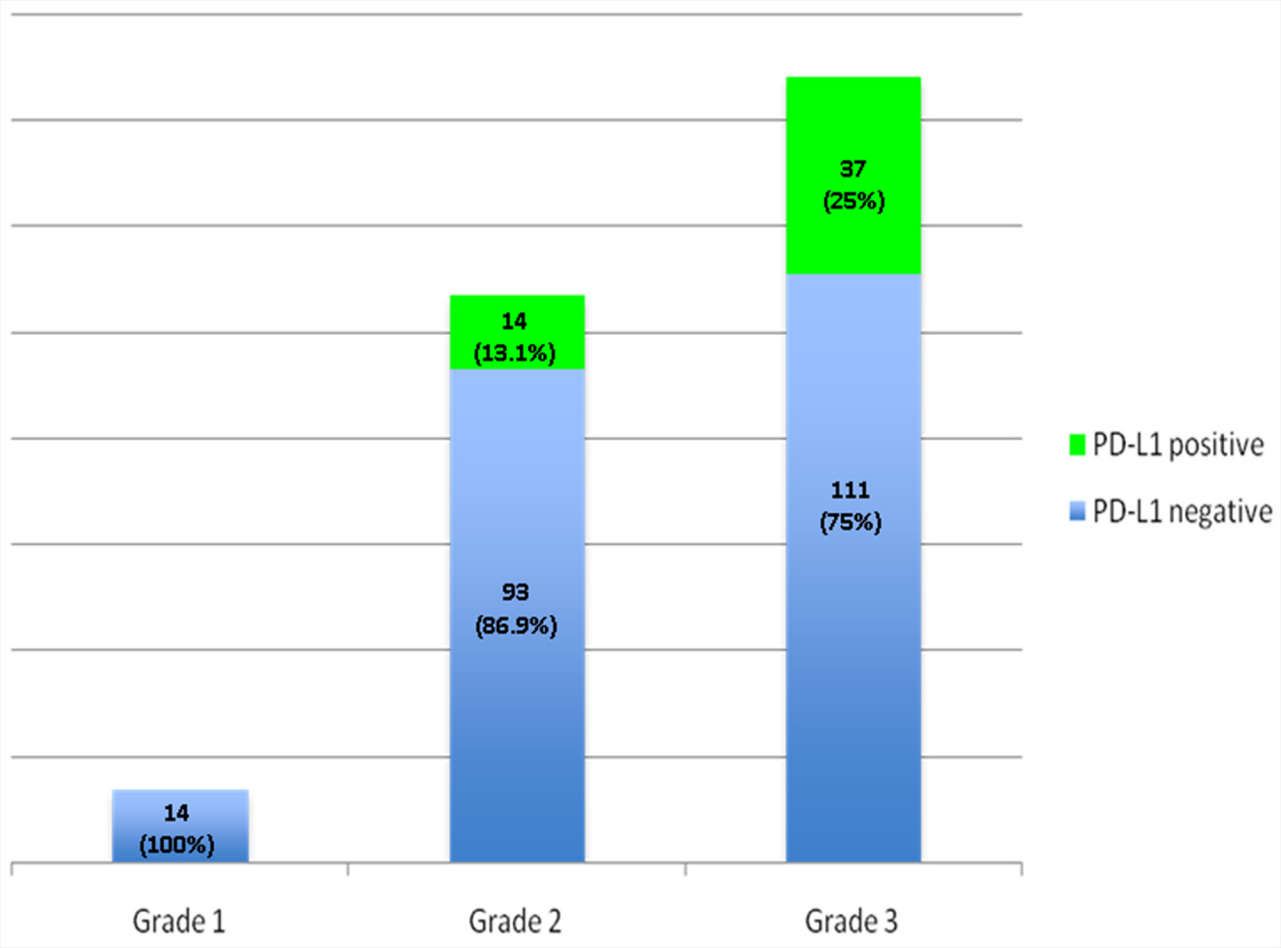
PD-L1 positivity correlation to tumor differentiation grade: StrongPD-L1 expression is statistically more common in grade 3 tumors compared to grade 1 and 2 tumors

Table 2 shows the correlation of PD-L1 status to clinical characteristics. No significant correlation to sex (p=0.64), smoking habit (p=0.17) and histology (p=0.34) was detected among the PD-L1 high, low and negative groups. Interestingly, there was a strong, statistically significant association of PD-L1 status with grading: strong PD-L1 expression was observed mostly among grade 3 tumors (72%), while only 27% of grade 2 tumors and no grade 1 tumor expressed PD-L1 at high levels (p=0.02). When the population was divided in PD-L1 positive and negative this correlation was even stronger: 25% of grade 3 tumors showed high levels of expression while only 13.1% of grade 1-2 tumors expressed the ligand (p=0.005, Figure 1).

**Table 2:** Correlation of PD-L1 status to clinical-pathological features

	PD-L1 <1% N (%)	PD-L1 1-49% N (%)	PD-L1 ≥50% N (%)	p value
**Gender**				p = 0.64
Male	139 (18.3%)	49	41 (81.7%)	
Female	37 (20%)	10	13 (80%)	
**Histology**				p = 0.34
Non-squamous	118 (63.1%)	37 (19.8%)	32 (17.1%)	
Squamous	49 (54.4%)	20 (22.2%)	21 (23.3%)	
**Grading**				**p = 0.02**
1+2	81 (66.9%)	26 (21.5%)	14 (11.6%)	
3	83 (56.1%)	28 (18.9%)	37 (25%)	
**Smoking status**				p = 0.17
Ever smoker	25 (62.5%)	10 (25%)	3 (33.3%)	
Never smoker	4 (44.4%)	2 (22.2%)	5 (12.5%)	

No association with any clinical characteristic was observed when the statistical analysis was performed considering PD-L1 expression as a continuous variable, with the only exception of tumor grading (p=0.02).

### Prognostic impact of PD-L1 expression

**Figure 2:**
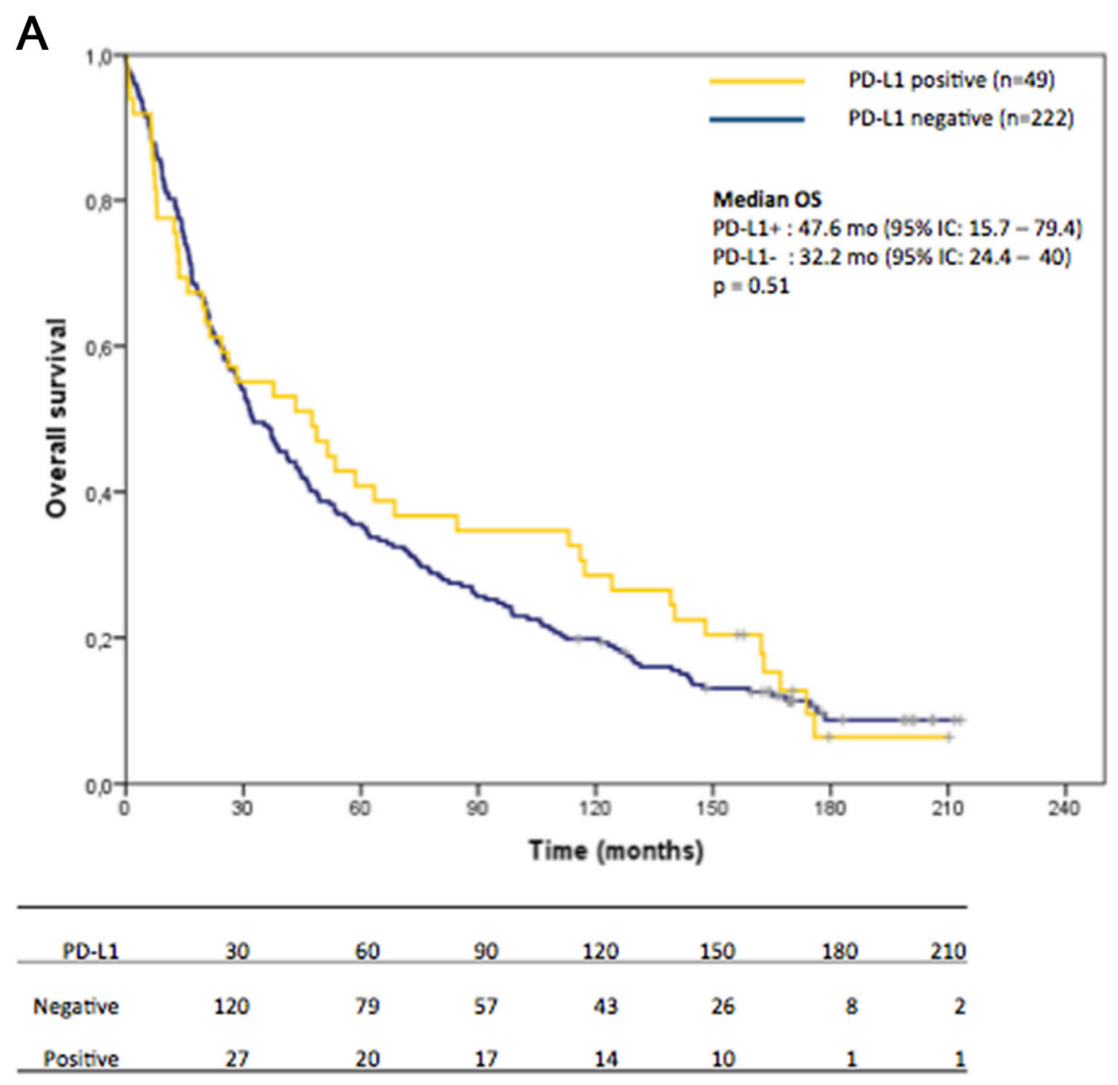

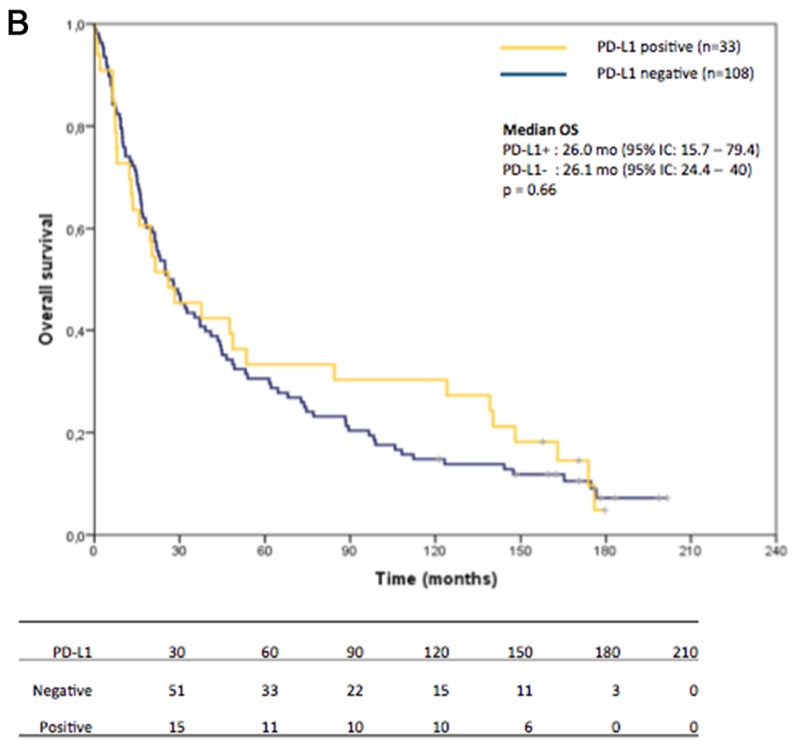

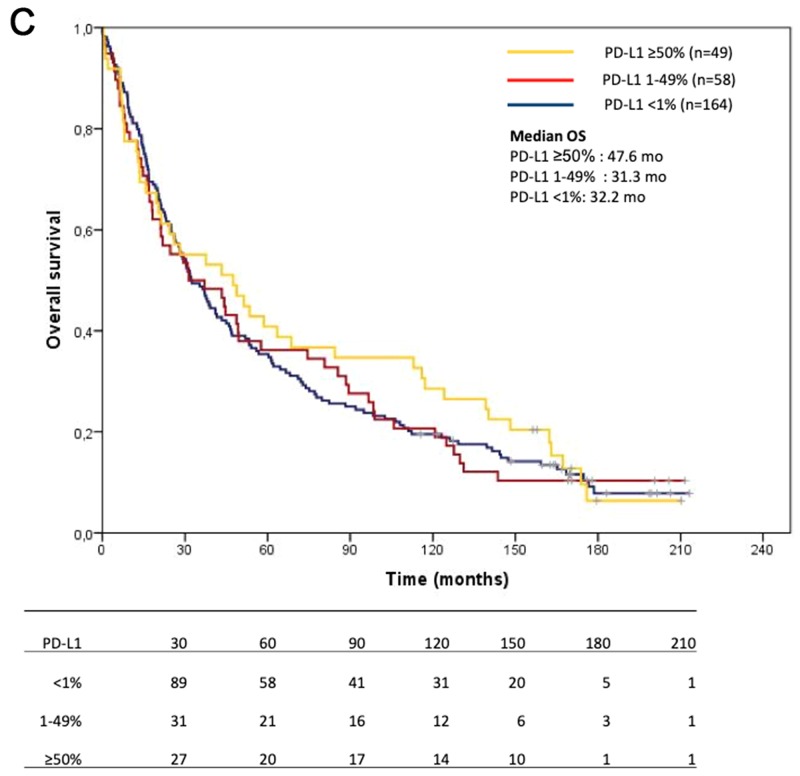

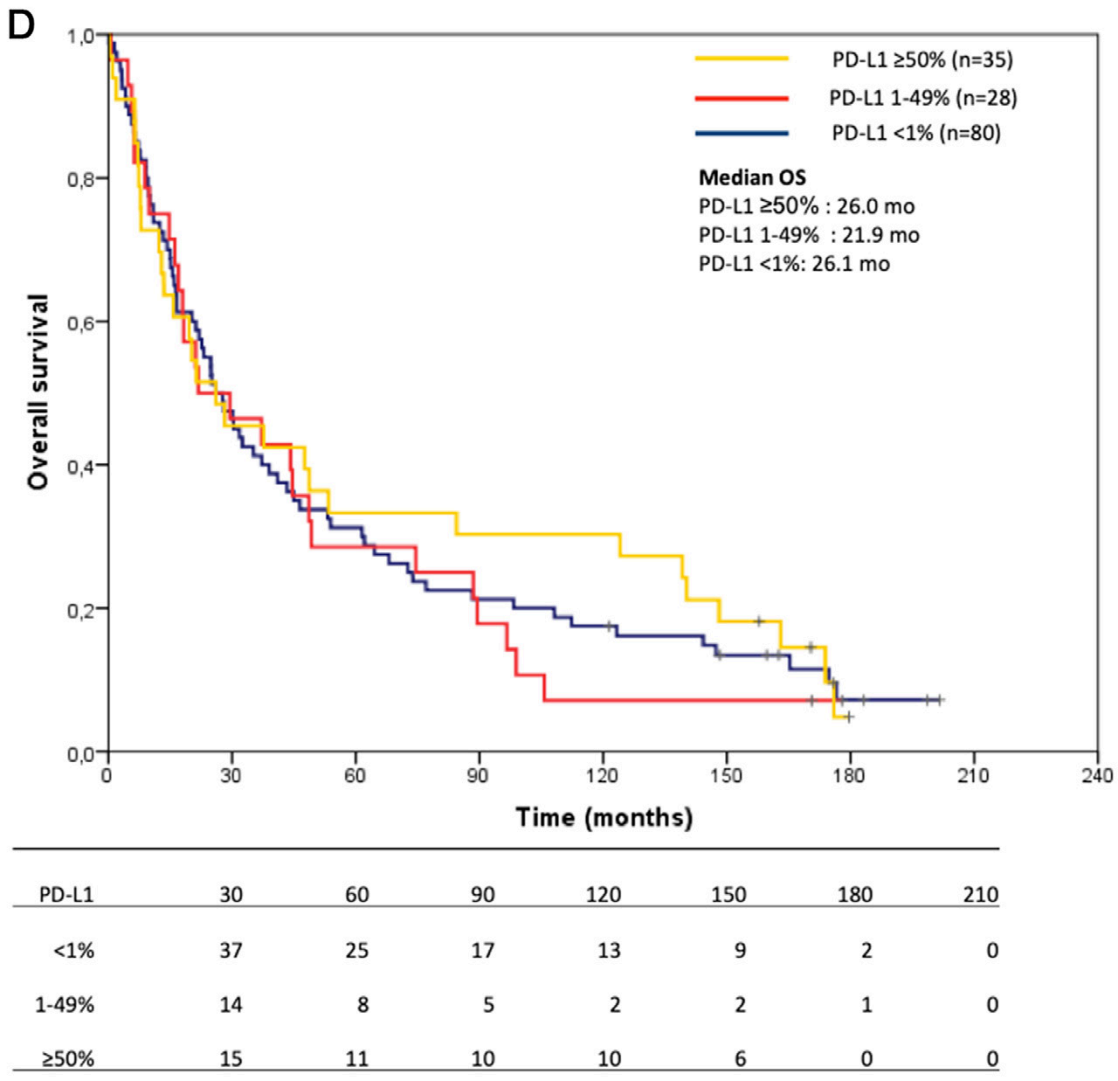
Kaplan-Meier curves of overall survival of the whole cohort **(A, B)** and grade 3 tumors **(C, D)**: No statistically significant difference in overall survival was observed between the several PD-L1 groups of patients.

OS data was available for 271 patients. No difference in overall survival (OS) was detected between PD-L1 positive (TPS ≥50%) and negative (TPS<50%) (PD-L1 positive vs negative: 47.6 vs 32.2 months, p=0.51, Figure 2A). When patients were divided in PD-L1 high (TPS ≥50%), low (TPS 1-49%) and negative (TPS<1%) we detected no correlation between PD-L1 status and survival (PD-L1 high vs low vs negative: 47.6 vs 31.3 vs 32.2 months, Figure 2B). Considering the strong association between tumor grade and PD-L1 expression, we restricted the analysis to patients with grade 3 tumors. Even in this subgroup of subjects, no difference in survival according to PD-L1 status was detected (Figure 2C and 2D).

## DISCUSSION

In the present study, specifically conducted in a NSCLC population not exposed to the potentially confounding effect of adjuvant chemotherapy, we showed that high levels of PD-L1 expression were not prognostic. Although PD-L1 expression was not associated with any specific clinical characteristic, we observed a strong association with tumor differentiation grade.

In NSCLC, biomarker analysis is not feasible in all patients mainly because of the scarce availability of tumor tissue. PD-L1 expression is not a perfect biomarker and much effort is being put in the research of more precise and reliable predictive markers for immunotherapy. An exploratory retrospective analysis of the CheckMate 026 study showed that high tumor mutation burden (TMB) identifies patients benefiting more from nivolumab than chemotherapy [49]. The same analysis detected no association between TMB status and PD-L1 expression [49]. No data are currently available on the association between TMB and differentiation grade, but it is possible that tumors with high somatic mutation burden are poorly differentiated. Support to this hypothesis comes from the recent observation of a higher incidence of *EGFR* mutations in well and moderately differentiated lung adenocarcinomas, likely as a reflection of lower TMB associated with presence of a single driver mutation [50]. The question arising from our observation is whether tumor differentiation grade could be useful as predictive marker of sensitivity to immunotherapy as this could have several advantages in daily clinical practice. The assessment of TMB requires high expertise, availability of tumor tissue and has a significant cost. On the other hand, the assessment of tumor differentiation grade is common pathology practice and requires no additional tumor tissue. Therefore, the possibility that tumor grade could help in defining patients potentially sensitive to immunotherapy deserves further investigation. At the same time, it is important to note that criteria for grading score should be better standardized.

In our study, no association of PD-L1 expression and survival was noted. Several other studies explored the prognostic impact of PD-L1 expression with conflicting results (Table 3). Interestingly, the only studies showing a negative impact of PD-L1 expression are those conducted in Asian populations, while all studies conducted in Caucasian patients showed no or a positive prognostic role of PD-L1 expression. Our study, including only Caucasian patients, confirmed that PD-L1 is not prognostic in surgically resected NSCLC. It is reasonable to suppose that the different prognostic effect detected in Caucasian versus Asian studies is related to the different biology of NSCLC in the two populations [51]. It is well known that the incidence of *EGFR* mutations and *KRAS* mutations is different in the two ethnic groups. *EGFR* mutations are present in 10-15% of Caucasian patients and up to 40% in Asians, while *KRAS* mutations are more frequently reported in Caucasians (20-30% versus <10% in Asians). Presence of *KRAS* mutations generally associates with worse prognosis, while patients harboring *EGFR* mutations have better outcome. In a recent study, Levy et al. showed that presence of *EGFR* mutations was associated with low tumor grade suggesting that the difference in prognosis observed among studies just reflects the different biology of the tumor in the two ethnic groups [51].

**Table 3:** Published studies on the prognostic significance of PD-L1 expression in NSCLC

Author	Year	Histology	Population	N° samples	PD-L1 Ab clone	PD-L1 positivity cut-off (%)	PD-L1 + (%)	Prognostic role (Pos/Neg/No)
Zhou C [12]	2017	Adenocarcinoma	Asian	108	SP263	H-score ≥1	40,7	Neg
Yvorel V [13]	2017	Sarcomatoid	Caucasian	36	E1L3N	≥5	75	Pos
Zhang M [14]	2017	Squamous	Asian	84	Abcam (28-8)	>5 (at least 2+)	58,3	Neg
Wu S [15]	2017	Adenocarcinoma	Asian	133	SP263	>25	16,5	Neg
Tsao M-S [16]	2017	All	Caucasian	982	E1L3N	≥1	32	No
						≥25	20,8	No
						≥50	14,3	No
Okita R [17]	2017	All	Asian	91	SP142	H-score ≥100	14	Neg
Igawa S [18]	2017	All	Asian	229	SP263	H-score ≥20	52	No
Takada K [19]	2017	Squamous	Asian	205	SP142	≥1	51,7	Neg
						≥5	35,1	No
						≥10	29,7	No
						≥50	18	No
Fend L [20]	2017	All	Caucasian	55	E1L3N	>5	27,3	No
Guo Q [21]	2017	Squamous	Asian	128	Ab58810	IRS≥3	61,7	Neg
Shimoji M [22]	2016	Adenocarcinoma	Asian	165	E1L3N	H-score ≥5	22	Neg
		Squamous		55			60	No
Sterlacci W [23]	2016	All	Caucasian	293	E1L3N	>5	12	Neg
Song Z [24]	2016	Adenocarcinoma	Asian	385	Proteintech	≥5	48,3	No
Ameratunga M [25]	2016	All	Caucasian	522	E1L3N	≥50	24	No (whole pop); Neg (EGFR+)
Inamura K [26]	2016	Adenocarcinoma	Asian	268	E1L3N	≥5	16	Neg
Mori S [27]	2017	Adenocarcinoma	Asian	296	EPR1611	Modified H-score ≥50	36	Neg
Sorensen SF [28]	2016	All	Caucasian	177	Ab58810	≥5	37,9	Pos
Vieira T [29]	2016	Sarcomatoid	Caucasian	75	B7H1	≥5	53	No
Yang CY [10]	2016	Squamous	Asian	105	NR	≥5	56,2	Pos
Sun JM [30]	2016	All	Asian	1070	E1L3N	≥1	45	No
						≥50	6	No
Dix Junqueira Pinto G. [31]	2016	All	Caucasian	177	Ab58810	≥5	32.8	No
Tang Y [32]	2015	NSCLC	Asian	170	E1L3N	H-score ≥5	65,9	No
Kim S [33]	2015	Squamous	Asian	331	E1L3N	≥10	26,9	No
Schimdt LH [34]	2015	All	Caucasian	321	E1L3N	≥5 (at least 2+)	24	No
Cooper WA [35]	2015	All	Caucasian	678	22C3	≥50	7,7	Pos
Lin C [36]	2015	Adenocarcinoma EGFRmut+	Asian	63	Ab58810	Mean IRS score	53,6	Pos
Velcheti V [9]	2014	All	Caucasian	155	5H1	> normal lung	36,1	Pos
				303			24,8	Pos
Zhang Y [37]	2014	Adenocarcinoma	Asian	143	SAB2900365	Quickscore>8	49	Neg
Yang CY [10]	2014	Adenocarcinoma	Asian	163	Proteintech	≥5	39,9	No
Azuma K [38]	2014	All	Asian	164	Lifespan Bioscience	H-score>30%	50	Neg
Chen YB [39]	2012	All	Asian	120	236A/E7	IRS≥3	57,5	Neg
Mu CY [40]	2011	All	Asian	109	NR	Mean H-score (NR)	53,2	Neg

In conclusion, this study shows that PD-L1 expression is not a prognostic factor in early-stage, surgically resected NSCLC. The strong correlation of PD-L1 expression with tumor grading suggests a potential role for tumor differentiation grade in selecting patients who will benefit more from immunotherapy.

## MATERIALS AND METHODS

### Ethics statement

The study (NCT03078959) was approved by the local ethical committee (Comitato Etico di IRST e Area Vasta Romagna – CEIIAV). All patients whose tumors were used for the purpose of this study had died at the time of sample collection/data analysis and there was no need for their written consent.

### Study objectives and patients

The primary objective of this retrospective study was to assess whether PD-L1 expression affects survival of stage I, II, III resectable non-small cell lung cancer. The cohort included 289 consecutive patients who underwent lung resection between 1997 and 2002. Main inclusion criteria for patient selection included availability of surgical tumor tissue and availability of clinical data including survival. Histology of all cases was revised according to the International Association for Lung Cancer Study/American Thoracic Society/European Respiratory Society (IASLC/ATS/ERS) classification for adenocarcinoma [52] and the 2004 World Health Organization (WHO) classification for other histologic type [53], based on p40 and TTF1 IHC staining. In order to guarantee tumor differentiation grading homogeneity, the grading score was reviewed by a pathologist (SD) at the time of collection of samples. Criteria used for the evaluation were based on evaluation of gland structure and nucleus characteristics (size, cromatin and nucleoli) according to recommendations of World Health Organization (WHO) [54, 55].

### Immunohistochemistry

Protein expression was evaluated by IHC on tissue micro-arrays (TMA) sections. Briefly, two cores of 2.0 mm were punched out from different areas of each formalin-fixed paraffin-embedded (FFPE) tumor block and randomly included in a TMA.

Unstained 4 μm tissue sections were dried in a drying oven at 60° for 1 hour. Slides were labeled with a bar-coded standardized antibody-specific protocol and loaded into a Benchmark XT® (Ventana Medical Systems, Inc) automated stainer. The primary antibody against PD-L1 (SP263, Ventana) was used according to the manufacturer instructions.

Two readers, a Pathologist (MP) and a Medical Oncologist (MD) with proven experience in IHC evaluation, independently performed IHC scoring. A score from 0 to 100 was assigned to each core according to the percentage of stained tumor cells. Immune-positivity was determined only by membrane staining, while cytoplasm staining and staining intensity were not taken into consideration for the purpose of this study. Cores with less than 100 tumor cells were considered not evaluable. In case of discordance of the scores of a single core, the cases were concurrently reviewed by the two readers in order to come to a consensus score.

The population of the study was then divided according to three criteria in 1) positive or negative according to a cut-off value of TPS of 50%; 2) as high (TPS ≥ 50%), low (TPS 1-49%) or negative (TPS <1%); 3) as a continuous variable.

### Statistical analysis

Clinical characteristics and associations with PD-L1 expression were examined with a descriptive analysis comparing the differences by χ2 test or Fisher’s exact test as appropriate. A p value of <0.05 was considered significant.

OS was evaluated according to PD-L1 status. OS was defined as the time between surgery and death. The Kaplan-Meier method was used with 95% confidence intervals. Comparison between groups was performed by log rank test. The significance level for all analyses was set at p<0.05 and all p-values were two-sided.

In a further analysis PD-L1 expression was considered as a continuous variable ranking from 0 to 100 and its correlation to clinical characteristics was investigated using the Mann-Whitney U test.

Statistical analysis was performed using IBM-SPSS Statistics version 20.

## Abbreviations

NSCLC: non-small cell lung cancer
PD-1: programmed death receptor 1
PD-L1: programmed death ligand 1
IHC: immunohistochemistry
EGFR: epidermal growth factor receptor
ALK: anaplastic lymphoma kinase
ROS-1: ROS protoncogene 1
OS: overall survival
KRAS: Kristen rat sarcoma virus
TMA: tissue microarray
FFPE: formalin-fixed paraffin-embedded
TMB: tumor mutation burden
TPS: tumor proportion score
